# 940. AI in Burn Depth Assessment: Systematic Review of Computational Approaches to a Longstanding Clinical Challenge

**DOI:** 10.1093/jbcr/irag033.507

**Published:** 2026-04-06

**Authors:** Christopher J Fedor, Bilal M Chaudhry, Natalie Carter, Jenny A Ziembicki, Francesco M Egro

**Affiliations:** University of Pittsburgh Medical Center, Department of Plastic Surgery, Pittsburgh, PA; Rutgers University, Newark, NJ; Herbert Wertheim College of Medicine, Miami, FL; University of Pittsburgh Medical Center, Pittsburgh, PA; University of Pittsburgh Medical Center, Pittsburgh, PA

## Abstract

**Introduction:**

Determining burn depth at the bedside remains one of the most persistent clinical challenges in burn care. Even expert burn surgeons, who rely on visual and tactile inspection, still misclassify up to 40% of cases. Uncertainty about burn depth, especially for lower TBSA cases, results in longer clinical observation impacting hospital costs and healing time/outcomes. Artificial intelligence (AI) has been investigated as a potential solution. While early efforts used classical machine learning (ML) models that depend on handcrafted color and texture features chosen by investigators, more recent convolutional neural networks (CNNs) have transformed the field by automatically learning features directly from images, enabling deeper, more accurate representations of burn severity.

**Methods:**

We systematically reviewed published studies applying AI to burn depth assessment. Of 49 included studies, we extracted information on dataset size, ground truth annotation, model type, classification granularity, and reported performance metrics. To highlight progress, accuracy values were grouped into three eras: classical ML, older CNNs, and newer CNNs.

**Results:**

Classical machine learning models achieved a mean accuracy of 79.6% (95% CI, 74.0–85.1%), with wide variability ranging from 55% to 97%. Older convolutional neural networks demonstrated less consistent performance, averaging 71.4% (95% CI, 63.1–79.6%). By contrast, newer CNNs achieved substantially higher and more reliable performance, with a mean accuracy of 91.9% (95% CI, 88.4–95.3%) and markedly narrower performance ranges.

Only one-third of studies explicitly distinguished superficial partial-thickness from deep partial-thickness burns, the most clinically critical boundary for grafting. Even in those that did, mean accuracy remained ~77–80%, with wider confidence intervals compared to the ~92% achieved by newer CNNs overall, emphasizing that the most clinically consequential distinction remains the most difficult to solve.

**Conclusions:**

Reported accuracies for AI in burn depth assessment are striking, with modern CNNs reaching 90–98%, seemingly far above the 60–70% accuracy historically observed among expert surgeons. Yet these values can be deceiving, as they often reflect dataset bias, surrogate ground truths, and simplified classification tasks rather than the most clinically critical distinctions. Nevertheless, the trajectory of newer CNNs demonstrates strong potential to advance burn assessment, highlighting the need for larger, more diverse image datasets to build generalizable AI systems capable of addressing real-world clinical challenges.

**Applicability of Research to Practice:**

Determining burn depth is critical for both TBSA calculation and operative decision-making, yet assessment remains highly subjective. Hospitals without burn centers stand to benefit most from AI-assisted tools, prompting the central question: where does this technology stand today?

**Funding for the study:**

N/A.

